# The first case report of kerion-type scalp mycosis caused by *Aspergillus protuberus*

**DOI:** 10.1186/s12879-019-4144-7

**Published:** 2019-06-10

**Authors:** Jinjing Jia, Min Chen, Xiumei Mo, Junfeng Liu, Fenggen Yan, Zhengxiao Li, Shaoqiong Xie, Dacan Chen

**Affiliations:** 1 Department of Dermatology, the Second Affiliated Hospital of Guangzhou University of Chinese Medicine, 111 Dade Road, Yuexiu District, Guangzhou, 510120 China; 2 Shanghai Key Laboratory of Molecular Medical Mycology, Department of Dermatology, Changzheng Hospital, Second Military Medical University, Shanghai, China; 3 Department of Dermatology, the Second Affiliated Hospital of Xi’an Jiaotong University, Xi’an, China; 4 Department of Dermatology, Shanghai Dermatology Hospital, 1278 Baode Road, Jing’an District, Shanghai, 200443 China

**Keywords:** Mycosis, Kerion, *Aspergillus protuberus*, Terbinafne

## Abstract

**Background:**

Scalp mycosis is often caused by dermatophytes and was so called tinea capitis. There is no published report caused by *Aspergillus protuberus*. We report a rare case of kerion-type scalp mycosis caused by *A. protuberus*.

**Case presentation:**

A 5-year-old girl developed pyogenic mass with pain for 8 days and got a fever for 2 days prior to admission. Surgical incision and drainage of the mass, intravenous cefuroxime and metronidazole in the local hospital aggravated the skin lesions. Species identification was performed by observation of morphologic and biochemical characteristicsand sequencing of the internal transcribed spacer (ITS) and β-tubulin (BT2). Treatment with oral and topical antifungal agents was effective with no relapse during the six months of clinical follow-up.

**Conclusions:**

*Aspergillus*is a opportunistic pathogenic fungus and its infection occurs mostly in patients with underlying conditions and immunocompromised statuses. So far no report of kerion-type scalp infection has been reported. The first case of kerion-type scalp mycosis caused by *A. protuberus* was described to highlight the importance of mycological examination that helps to recognize rare pathogenic fungi. Any boggy lesion with hair loss over the scalp and non-responsive to antibiotics should be suspected as resulting from fungal infection, and mycological examination should be performed, especially in children.

## Background

Kerion is a type of tinea capitis(TC) often caused by zoophilic dermatophytes. *Aspergillus protuberus* is a species of fungus belonging to the genus *Aspergillus*; it was previously considered a member of the section *Versicolores* that can cause opportunistic infections in immunocompromised patients. However, *A. protuberus* has recently been described as a separate species [1]. Although the members of *Aspergillus* section *Versicolores* have been implicated in rare cases of lung, eye, and nail infections [2–4], there is no published report of human scalp mycosis caused by *A. protuberus*. Here, we report a case of kerion-type scalp mycosis that was identified as being caused by *A.protuberus* infection through mycological examination.

## Case presentation

A 5-year-old girl presented with a pyogenic mass and pain of the scalp for 8 days, plus fever for 2 days. Surgical incision and drainage of the mass was performed, and cefuroxime and metronidazole was administered intravenously in the local hospital, but there was no obvious improvement. The skin lesions gradually increased, part of which formed an ulcer surface, and the purulent secretion increased. A fever began 2 days prior to admission, with a highest temperature of 39 °C. So, she came to our clinic for further diagnosis and treatmenton February 12, 2018. The patient was living in the countryside and had a history of dog contact; however, she was too young to recall a history of trauma. She was normally healthy with no similar diseases, other infectious diseases, or genetic diseases in her family.

**Fig. 1:**
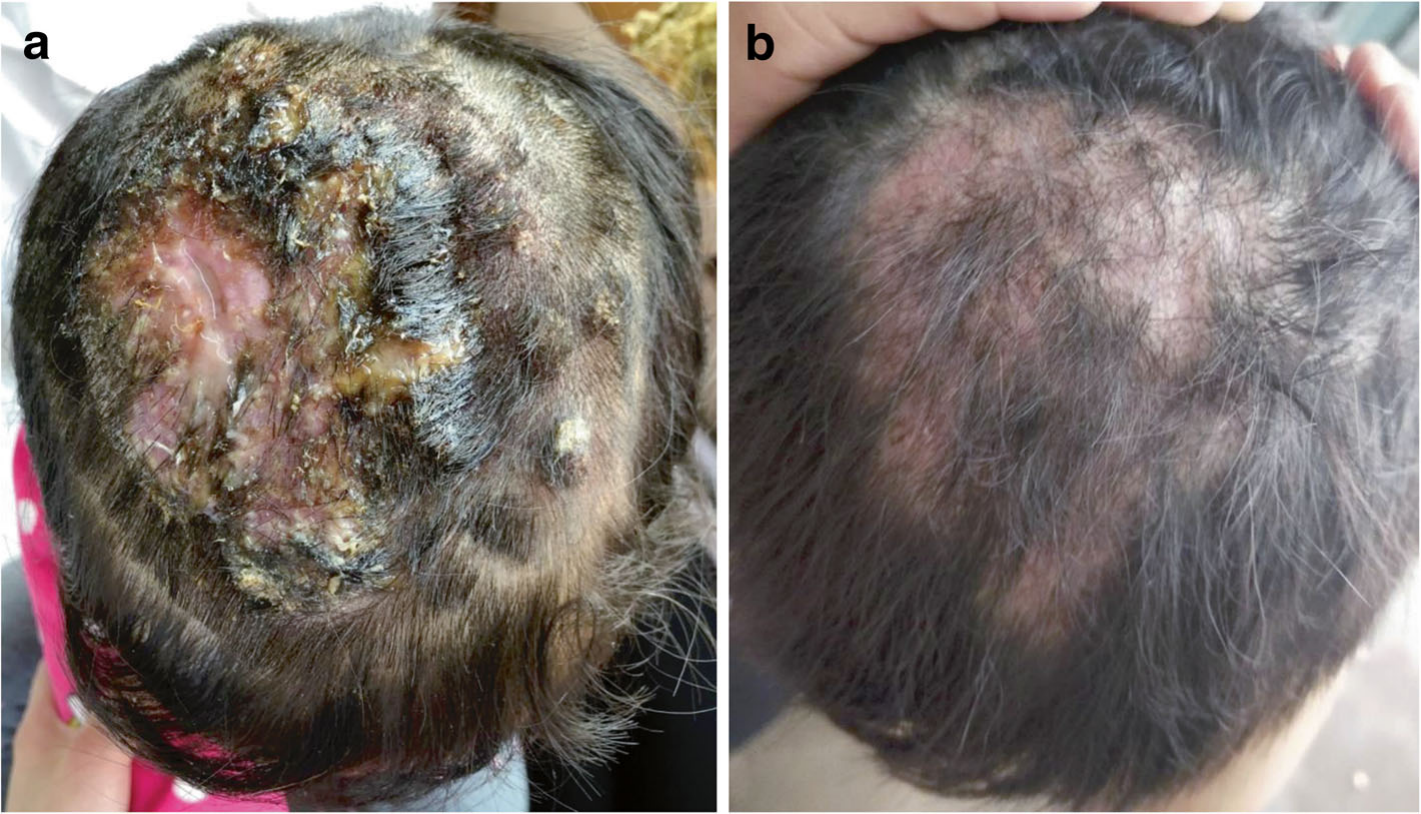
**a** A large 10 × 12 cm sized erythematous boggy swelling over the scalp with several ulcers and hair loss. **b** After 70 days of treatment,the lesions were resolved, leaving residual scarring alopecia

Cutaneous examination revealed several ulcers of different sizes fused into a large 10 by 12 cm tender erythematous boggy swelling over the scalp with significant loss of hair, and yellowish-brown to hemorrhagic crusts. Removal of the crusts revealed seropurulent discharge. There was obvious stench and tenderness in the lesions (Fig. 1a).

A routine blood test showed the white blood cell count to be 12.41 × 10^9^/L (4–10^9^/L), consisting of 8.90 × 10^9^/L (2–7 × 10^9^/L) neutrophils at a percentage of 71.70% (25–60%). The erythrocyte sedimentation rate (ESR) was 43 mm/h (0–20 mm/h). Routine urine, fecal, liver function, and renal function examinations revealed no obvious abnormalities. Bacterial culture yielded growth of *Staphylococcus aureus.*

**Fig. 2:**
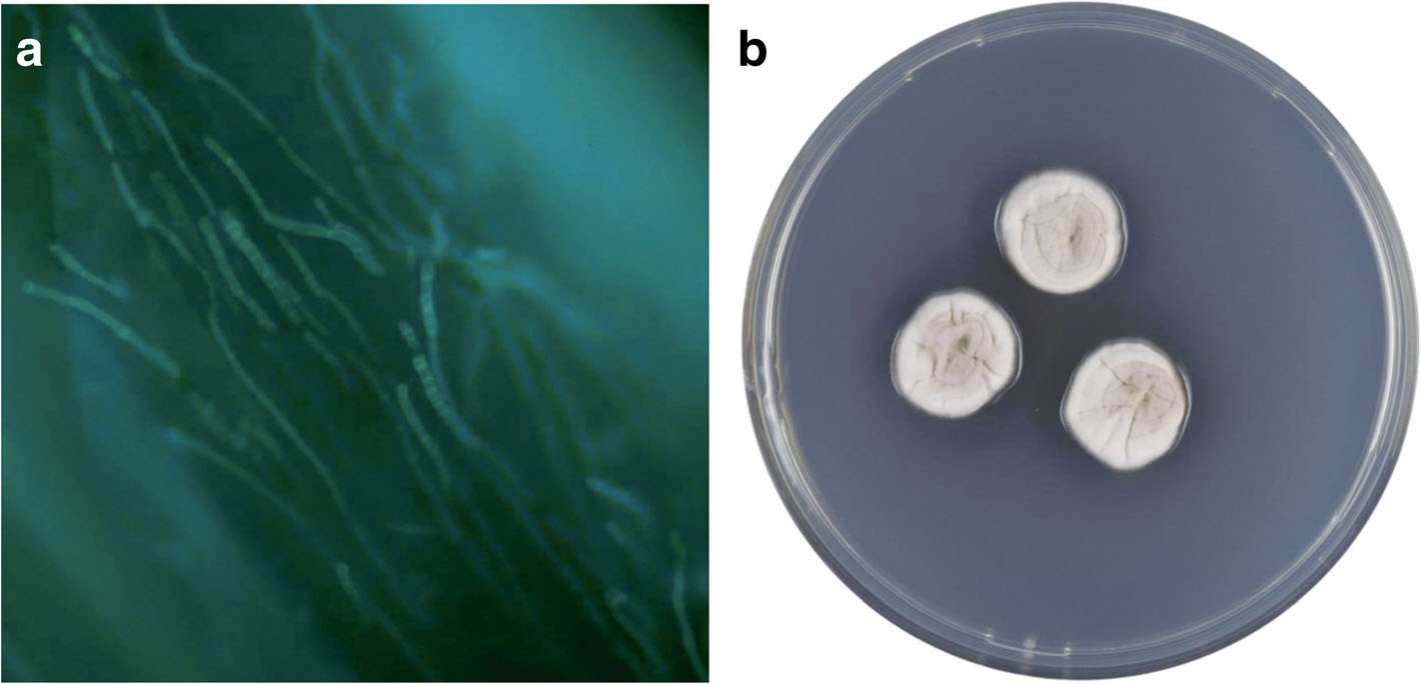
**a** Fungal fluoroscopy showed septate hyphae inside the hair root. **b** Black and hairy colonies in 15 d

Affected hair and excretion from the ulcer were collected and prepared for fluorescent brightening agents and Evans blue staining using a 10% potassium hydroxide (KOH) solution. We found fungi with septate hyphae inside the hair root (Fig. 2a). These samples were then inoculated onto Sabouraud agar (OXOID, Inc., Basingstoke, Hampshire, U.K.) and Czapek’s agar (OXOID, Inc., Basingstoke, Hampshire, U.K) and incubated at 30 °C. Growth was apparent within 15 days on all agar plates. The colonies were initially gray and fluffy and spread rapidly; eventually, the colonies became hairy and reached a diameter of 8 cm (Fig. 2b).

**Fig. 3:**
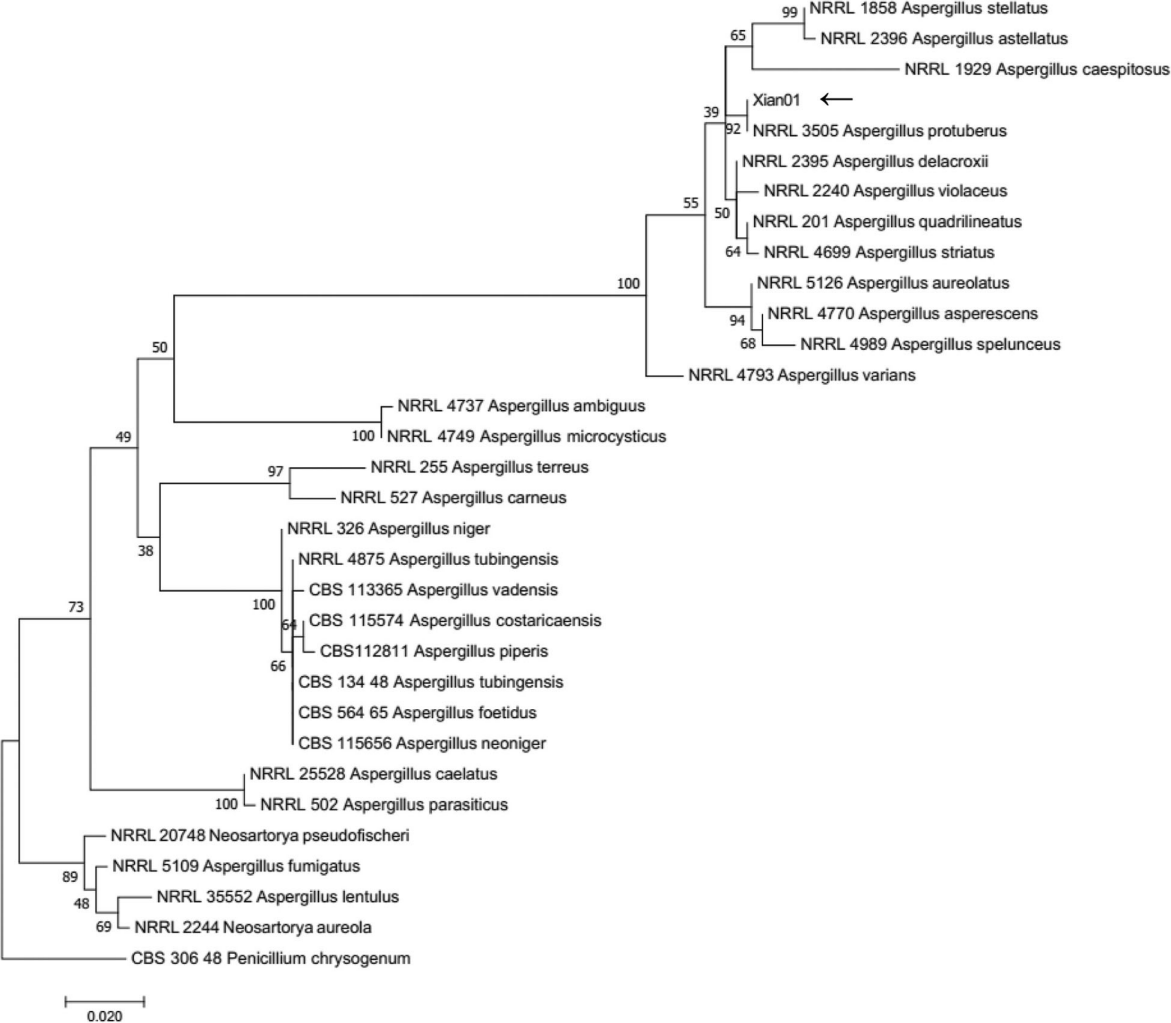
Phylogenetic analysis of *A.protuberus*(isolate of the present case temporary named Xian01). Phylogenetic tree resulting from maximunlikehood (ML) and neighbor-joining (NJ) analysis for the ITS and BT2 genes

DNA was extracted following the Quick CTAB and PCR protocols as described previously [5]. The sequences of ribosomal internal transcribedspacer (ITS) and β-tubulin (BT2)were amplified (Table 1). DNA from each isolate was amplified by PCR in 12.5 ml reaction volumes using the primers and protocols described previously [6]. Blast results of the sequences in GenBank revealed that ourisolate belonged to *Aspergillusprotuberus*with 99–100% similarity to depositeditems.The phylogenetic tree was made using MEGA v. 7.0.3.Phylogenetic analysis of concatenated loci ITS and BT2 showed that the reported clinical isolate (Temporarynamed Xian01) was definitivelynested within the *A.protuberus* species cluster (Fig. 3).

**Table 1:** Primers and PCR amplification conditions used for molecular identification

Locus	Primers	Primer sequence	Amplification conditions
*ITS*	ITS5	5′-GGAAGTAAAAGTCGTAACAAGG-3′	94 °C 5 min; 30 cycles: 94 °C 60s, 55 °C 60s, 72 °C 60s; 72 °C 10 min
	ITS4	5′-TCCTCCGCTTATTGATATGC-3′	
*BT2*	BT2a	5′-GGTAACCAAATCGGTGCTGCTTTC-3′	95 °C 5 min; 35 cycles: 95 °C 30s, 58 °C 30s, 72 °C 1 min; 72 °C 7 min
	BT2b	5′-ACCCTCAGTGTAGTGACCCTTGGC-3′

The patient was diagnosed with scalp mycosis caused by*A.protuberus*, and secondary *S.aureus* infection. She began a treatment course oforal terbinafine (125 mg/day, 14 days) and methylprednisolone(8 mg/day, 5 days), intravenous mezlocillin sodium, sulbactam sodium (1.875 g/day, 7 days), compound glycyrrhizin (20 ml/day, 14 days), a wet dressing of ethacridine lactate and topical naphthotifen ketoconazole ointment, and fusidic acid cream. After the above described treatment, all the original lesions formed scabs without purulent secretions. Fluorescence microscopy for fungal detection showed negative result. The patient continued treatment with oral terbinafine (125 mg/day, 42 days that was decreased to 125 mg/two days, 14 days) and compound glycyrrhizin(25 mg/day, 56 days), with topical naphthotifen ketoconazole ointment. This treatment regimen cured the patient without adversely affecting liver function or resulting in other adverse drug-related events. Most of the patient’s hair regrew with only small areas of alopecia left (Fig. 1b). No relapse was observed during the 6 months of follow-up.

## Discussion and conclusions

Scalp mycosis is often caused by dermatophytes is also referred to as TC. It is mainly observed in school-going children, and rarely in adults. Close contact with patients with TC, animals, and contaminated objects are the main routes of infection [7]. It can also occur in adults with diabetes, infection, organ transplantation, and other immunosuppressive conditions, and in individuals taking immunosuppressive drugs [8]. Kerion represents an inflammatory variant of TC caused by a dramatic immune response to fungus [9, 10]. The lesion begins as a group of inflammatory follicular papules, gradually fusing into a protuberant inflammatory mass with a soft texture. The surface then transforms into honeycomb-shaped pus-discharging pores. Secondary bacterial infection leads to abscess formation [11, 12]. This type of infection can destroy hair follicles, thus potentially leading to permanent alopecia and scar formation. Therefore, early diagnosis and timely treatment are very important. However, due to the obvious inflammation of the hair follicle and the surrounding area, it is often misdiagnosed and treated as scalp pyoderma, carbuncle,or cellulitis. Incision and drainage aggravate the infection and lead to a delay in treatment of the disease. Therefore, any boggy lesion with hair loss over the scalp and non-responsive to antibiotics should be suspected as fungal infection and be subjected to mycological examination, especially in children [13].

The etiological agents responsible for causing kerion are most commonly zoophilic dermatophytes such as *Microsporum canis* and *Trichophyton violaceum* [9, 10]. To our knowledge, there are no published reports of kerion-type scalp mycosis caused by *A.protuberus*. In this case, the patient had a history of contact with dogs and lived in rural areas with poor hygienic conditions. Although the medical history is short, the disease was aggravated by misdiagnosis and inappropriate incision and drainage at local hospitals, with severe infections and inflammatory reactions that were complicated by *S. aureus* infection. Fortunately, fungal infection was promptly detected and diagnosed. However, it is unclear why *A. protuberus*, not the common zoophilic dermatophyte, caused this infection. Borsa as reported a case of vaginitis caused by *A. protuberus*. To the best of our knowledge, there is no other published case of human infection caused by this species [14]. Other members of *Aspergillus* section *Versicolores* have been found in rare cases of lung [2], eye [3, 4], ear [15], bone [16], and nail [17] infections, mainly in patients with other underlying conditions and immunocompromised statuses. The patient in this case report was in good physical condition without any systemic diseases or immunosuppressive status. Apart from the contact with dogs, frequent exposure to soil and crops and careless injuries while playing were not excluded as routes of transmission. However, the demographic characteristics, probable origin, and route of transmission still need further investigation.

As for the treatment, itraconazole, fluconazole, and amphotericin B performed poorly in deep infections of *A. protuberus* [2, 4]. However, in superficial infection, terbinafine, itraconazole, and voriconazole were all effective [3, 15, 17]. Although griseofulvin is the traditional “gold standard” for general TC, its side effects limit its use at present [8, 18]. New antifungal agents, itraconazole and terbinafine, are widely used nowadays; however, itraconazole is rarely used in children due to limited clinical data. Only when the advantages outweigh the disadvantages, it can be cautiously used in children. Terbinafine, a lipophilic and keratophilic agent, has high concentrations in skin, hair, and nails, and has broad-spectrum antifungal activity (against dermatophytes, yeast, *Aspergillus* species, *Histoplasma capsulatum* etc.). It can be used in children over 2 years old, so it is often used as a first-line medication for TC in children [18]. Pediatric dosages are weight-based, at 62.5 mg (10–20 kg) or 125 mg (20–40 kg) daily. Hepatotoxicity is the main adverse reaction of the systemic use of antifungal drugs. Therefore, liver function should be monitored during their use. When transaminase is slightly elevated, liver protectants should be given timely. When liver function damage is serious, it is necessary to stop treatment. The standard of clinical cure is subsidence of skin lesions, but fungal reexaminations every 2–4 weeks are recommended, because mycological cure is the gold standard [19]. Although there is no standard treatment for kerion-type scalp mycosis caused by *A. protuberus* in the literature, we still referred to the general therapeutic schedule for TC. Because of its strong reaction to allergy similar to kerion, we adopted a short course of oral corticosteroids to reduce inflammation and the possibility of permanent scarring or alopecia. In addition, because the patient had a secondary bacterial infection, we also gave her antibiotics at the initial treatment. The therapeutic effect was good and resulted in mycological cure. Most of her hair regrew with no relapse during the six months’ follow-up.

To our knowledge, we report the first case of kerion-type scalp mycosis caused by *A. protuberus*; this report was supported by clinical, microbiological, and molecular data. It reminds clinicians that besides general dermatophytes, novel fungal infectious agents can also cause kerion-type scalp mycosis. Mycological examination is a useful tool to recognize pathogens. Any boggy lesions with hair loss over the scalp and non-responsive to antibiotics should be suspected as resulting from fungal infection, and mycological examination should be performed, especially in children.

## Data Availability

Not applicable.

## Abbreviations

*Aspergillusprotuberus*: *A. protuberus*
BT2: β-tubulin
ESR: Erythrocyte sedimentation rate
ITS: Internal transcribed spacer
KOH: Potassium hydroxide
*Staphylococcus aureus*: *S.aureus*
TC: Tineacapitis

## References

[1] Siqueira JP, Sutton DA, García D, Gené J, Thomson P, Wiederhold N, Guarro J (2016). Species diversity of Aspergillus section Versicolores in clinical samples and antifungal susceptibilityFungal. Biol..

[2] Charles MP, Noyal MJ, Easow JM, M R (2011). Invasive pulmonary aspergillosis caused by Aspergillusversicolor in a patient on mechanical ventilation. Australas Med J.

[3] Al-Hatmi AMS, Castro MA, de Hoog GS, Badali H, Alvarado VF, Verweij PE, Meis JF, Zago VV (2019). Epidemiology of Aspergillus species causing keratitis in Mexico. Mycoses..

[4] Perri P, Campa C, Incorvaia C, Parmeggiani F, Lamberti G, Costagliola C, Sebastiani A (2005). Endogenous Aspergillusversicolorendophthalmitis in an immuno-competent HIV-positive patient. Mycopathologia.

[5] Al-Hatmi AM, van Diepeningen AD, Curfs-Breuker I, de Hoog GS, Meis JF (2015). Specific antifungal susceptibility profiles of opportunists in the Fusariumfujikuroi complex. J AntimicrobChemother.

[6] Stielow JB, Lévesque CA, Seifert KA, Meyer W, Iriny L, Smits D, Renfurm R, Verkley GJ, Groenewald M, Chaduli D, Lomascolo A, Welti S, Lesage-Meessen L, Favel A, Al-Hatmi AM, Damm U, Yilmaz N, Houbraken J, Lombard L, Quaedvlieg W, Binder M, Vaas LA, Vu D, Yurkov A, Begerow D, Roehl O, Guerreiro M, Fonseca A, Samerpitak K, van Diepeningen AD, Dolatabadi S, Moreno LF, Casaregola S, Mallet S, Jacques N, Roscini L, Egidi E, Bizet C, Garcia-Hermoso D, Martín MP, Deng S, Groenewald JZ, Boekhout T, de Beer ZW, Barnes I, Duong TA, Wingfield MJ, de Hoog GS, Crous PW, Lewis CT, Hambleton S, Moussa TA, Al-Zahrani HS, Almaghrabi OA, Louis-Seize G, Assabgui R, McCormick W, Omer G, Dukik K, Cardinali G, Eberhardt U, de Vries M, Robert V (2015). One fungus, which genes? Development and assessment of universal primers for potential secondary fungal DNA barcodes. Persoonia..

[7] Kechia FA, Kouoto EA, Nkoa T, Nweze EI, Fokoua DC, Fosso S, Somo MR (2014). Epidemiology of tineacapitis among school-age children in Meiganga, Cameroon. J Mycol Med.

[8] Hillary T, Suys E (2014). An outbreak of tineacapitis in elderly patients. Int J Dermatol.

[9] Kudava K, Kituashvili T, Sekania M, Galdava G (2013). Some characteristics of tineacapitis. Iran J Pediatr.

[10] Sonthalia S, Khurana R (2016). Kerion. Indian J Pediatr.

[11] Elewski BE (2000). Tineacapitis: a current perspective. J Am AcadDermatol.

[12] Zaraa I, Hawilo A, Aounallah A, Trojjet S, El Euch D, Mokni M, Ben Osman A (2013). Inflammatory Tineacapitis: a 12-year study and a review of the literature. Mycoses..

[13] von Laer Tschudin L, Laffitte E, Baudraz-Rosselet F, Dushi G, Hohlfeld J, de Buys Roessingh AS (2007). Tineacapitis: no incision nor excision. J PediatrSurg.

[14] Borsa BA, Özgün G, Houbraken J, Ökmen F (2015). The first case of persistent vaginitis due to Aspergillusprotuberus in an immunocompetent patient. Mikrobiyol Bul.

[15] Rotoli M, Sascaro G, Cavalieri S (2001). Aspergillusversicolor infection of the external auditory canal successfullytreated with terbinafne. Dermatology.

[16] Liu Z, Hou T, Shen Q, Liao W, Xu H (1995). Osteomyelitis of sacral spine caused by Aspergillusversicolor with neurologic defcits. Chin Med J.

[17] Veraldi S, Chiaratti A, Harak H (2010). Onychomycosis caused by Aspergillusversicolor. Mycoses.

[18] Kakourou T, Uksal U (2010). European Society for Pediatric Dermatology.Guidelines for the management of tineacapitis in children. PediatrDermatol..

[19] Patel GA, Schwartz RA (2011). Tineacapitis: still an unsolved problem?. Mycoses..

